# Comparative Metabolomic and Transcriptomic Analyses Reveal Distinct Ascorbic Acid (AsA) Accumulation Patterns between PCA and PCNA Persimmon Developing Fruit

**DOI:** 10.3390/ijms242015362

**Published:** 2023-10-19

**Authors:** Yiru Wang, Songfeng Diao, Huawei Li, Lingshuai Ye, Yujing Suo, Yanhao Zheng, Peng Sun, Weijuan Han, Jianmin Fu

**Affiliations:** 1 Research Institute of Non-Timber Forestry, Chinese Academy of Forestry, Zhengzhou 450003, China; yiruwang@caf.ac.cn (Y.W.); dsf@caf.ac.cn (S.D.); lihuawei@caf.ac.cn (H.L.); yes@caf.ac.cn (L.Y.); suoyj@caf.ac.cn (Y.S.); zyh@caf.ac.cn (Y.Z.); ptsunpeng@caf.ac.cn (P.S.); 2 Research Institute of Fast-Growing Trees, Chinese Academy of Forestry, Zhanjiang 524022, China

**Keywords:** *Diospyros kaki*, ascorbic acid, astringency type, fruit developing stage

## Abstract

Persimmon fruit has a high nutritional value and significantly varies between pollination-constant astringent (PCA) and pollination-constant non-astringent (PCNA) persimmons. The astringency type affects sugar, flavonoids, and tannin accumulation and is well known in persimmon fruit. However, the impact of the fruit astringency type on ascorbic acid (AsA) accumulation is limited. In this study, typical PCA varieties (‘Huojing’ and ‘Zhongshi5’) and PCNA varieties (‘Yohou’ and ‘Jiro’) of persimmon fruit were sampled at four developing stages (S1–S4) to provide valuable information on AsA content variation in PCA and PCNA persimmon. Persimmon fruit is rich in ascorbic acid; the AsA content of the four varieties ‘Zhongshi5’, ‘Huojing’, ‘Jiro’, and ‘Youhou’ mature fruit reached 104.49, 48.69, 69.69, and 47.48 mg/100 g. Fruit of the same astringency type persimmon showed a similar AsA accumulation pattern. AsA content was significantly higher in PCA than PCNA fruit at S1–S3. The initial KEGG analysis of metabolites showed that galactose metabolism is the major biosynthetic pathway of AsA in persimmon fruit. There were significant differences in galactose pathway-related metabolite content in developing PCA and PCNA fruit, such as Lactose, D-Tagatose, and D-Sorbitol content in PCA being higher than that of PCNA. Combined gene expression and WGCNA analyses showed that the expression of the *GME* (*evm.TU.contig4144.37*) gene was higher in PCA-type than in PCNA-type fruit in S1–S3 and exhibited the highest correlation with AsA content (r = 690 **, *p* < 0.01). Four hub genes, including the *DNA methylation gene*, *methyltransferase gene*, *F-box*, and *Actin-like Protein*, were identified as potential regulators of the *GME* gene. These results provide basic information on how astringency types affect AsA accumulation and will provide valuable information for further investigation on AsA content variation in persimmon fruit.

## 1. Introduction

Ascorbic acid (AsA) is a natural water-soluble vitamin (Vitamin C) that is involved in the prevention of humans from various oxidative stress-related diseases [1,2,3,4]. Humans cannot synthesize AsA because the L-gulono-1,4-lactone oxidase is non-functional in the AsA biosynthesis pathway [5]. Plant foods are the main source of AsA for humans in their diet because humans cannot synthesize AsA [6,7,8]. In plants, AsA is present in high quantities in almost all subcellular compartments as well as in the apoplast and participates in a variety of biological processes, including photosynthesis, photoprotection, resistance to environmental stresses, floral induction, seed germination, senescence, hormone biosynthesis, and ROS regulation [1,2,9,10]. In plants, AsA biosynthesis occurs through the L-galactose pathway and other alternative pathways, including the myo-inositol pathway, L-gulose pathway, and D-galacturonate pathway [11]. The L-galactose pathway has been reported to be the main pathway for AsA biosynthesis in various plant species, in which ascorbic acid is synthesized from GDP-D-mannose to GDP-L-galactose [12,13]. Genes encoding enzymes required for this pathway have been identified and cloned in plants [1]. In addition, the regeneration of ascorbic acid through the Foyer–Halliwell–Asada cycle also plays an essential role in maintaining AsA content [14,15]. AsA levels vary significantly among different plants and at different development stages [16,17,18].

Persimmon (*Diospyros kaki* Thunb.) is a plant in the family Ebenaceae with a long cultivation history, also known as a woody grain [19]. As an essential fruit tree in Asian countries, persimmon has become increasingly popular with its high commercial value and health benefits for humans [20]. Persimmon fruits are usually classified into four types based on the mode of fruit astringency loss: pollination-constant non-astringent (PCNA), pollination variant non-astringent (PVNA), pollination-constant astringent (PCA), and pollination-variant astringent (PVA) [21]. Nearly all cultivars belong to the PCA and PCNA types. The quality of persimmon fruit is highly variable, with significant differences between PCNA and PCA persimmon [19,22]. In persimmon fruit, it is well known that types of astringency influence the concentration of proanthocyanidins, sugars, and flavonoids [19,23]. However, very limited information is currently available about the impact of the fruit astringency type on the AsA metabolic pathway metabolites and their associated genes in persimmon. 

A transcriptome is the complete set of transcriptional information by certain cells or tissues at specific developmental stages or under physiological conditions [24]. Metabolites are the final result of gene transcription in an organism under internal and external regulation and are the material basis of the phenotype [25,26]. Transcriptomic combined with metabolomic analyses can screen key metabolic pathways, genes, and metabolites more systematically and comprehensively for subsequent research and application [27,28]. Omics analysis methods such as metabolome and transcriptome can provide abundant data information, and different conclusions can be drawn from different perspectives. In this study, a re-analysis of certain metabolic and transcriptomic data [19,22] along with new data was conducted to further investigate AsA metabolic pathway gene expression and metabolite accumulated profiles in developing PCA and PCNA persimmon fruit. These results will provide valuable information on AsA content variation in persimmon fruit and further provide useful information for further investigation on how astringency types affect ascorbic acid accumulation between PCA and PCNA persimmon fruit.

## 2. Results

### 2.1. Content of AsA in Persimmon Fruit

**Figure 1:**
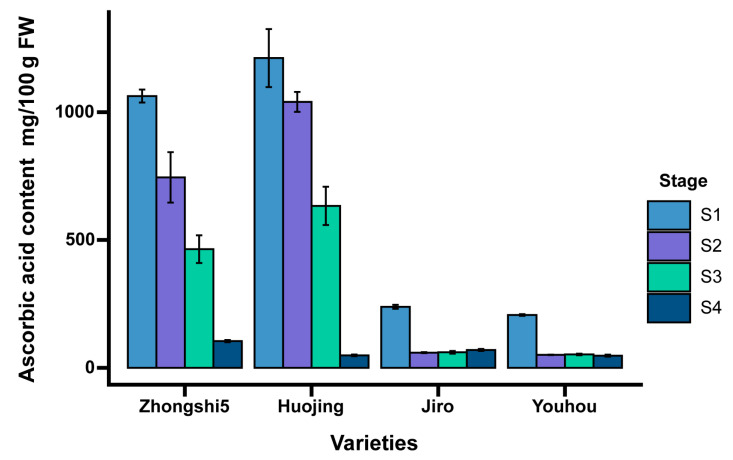
Fruit AsA content at four developmental stages in four persimmon varieties. Four developmental stages included the young fruit stage (S1), fruit expansion stage (S2), turning stage (S3), and mature stage (S4). Values were the means of three replicates ± SD, and the error bars represent the standard deviation.

AsA accumulation patterns at four different stages were different in different astringency-type persimmon fruit, including PCA-type persimmon varieties (‘Huojing’ and ‘Zhongshi5’) and PCNA-type varieties (‘Yohou’ and ‘Jiro’). Four typical developing stages, including the young fruit stage (S1), fruit expansion stage (S2), turning stage (S3), and mature stage (S4), were selected for further investigation. The AsA contents of the four varieties ‘Zhongshi5’, ‘Huojing’, ‘Jiro’, and ‘Youhou’ mature fruit reached 104.49, 48.69, 69.69, and 47.48 mg/100 g of fresh weight (FW), respectively, indicating that the persimmon fruit is rich in ascorbic acid (Figure 1). There was an overall decreasing trend in the AsA content of persimmon fruit throughout the developmental stages.

Fruit of the same astringency type persimmon shows similar patterns of ascorbic acid accumulation. Ascorbic acid content was significantly higher in PCA persimmon fruit than in PCNA fruit at the young fruit stage, fruit expansion stage, and turning stage. A significant and continuous reduction of AsA content was observed during fruit development in ‘Zhongshi5’ and ‘Huojing’. At the same time, the AsA values in ‘Jiro’ and ‘Yohou’ were substantially decreased at S2, followed by a nearly stable content. The highest concentration of AsA was detected at S1 in ‘Huojing’ (1211.7 mg/100 g FW), whereas the lowest content was observed at S4 in ‘Yohou’ (47.48 mg/100 g FW).

### 2.2. Metabolic Features of PCA and PCNA Persimmon Developing Fruit

**Figure 2:**
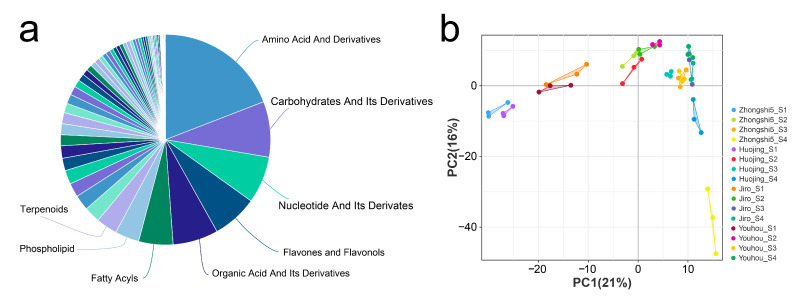
Comparison of metabolites in four development stages of PCNA (‘Jiro’ and ‘Youhou’) and PCA (‘Zhongshi5’ and ‘Huojing’) fruit. (**a**) Statistics on the major categories of metabolites. (**b**) Principal component analysis of metabolites.

To compare the metabolite accumulated profile between PCNA (‘Jiro’ and ‘Youhou’) and PCA (‘Zhongshi5’ and ‘Huojing’) persimmon developing fruit, metabolite compositions were evaluated using quasi-targeted metabolomics (Table S1). A total of 889 metabolites of eight major categories were identified in four development stages of PCNA and PCA fruit, including amino acid and derivatives (170), carbohydrates and their derivatives (76), nucleotides and their derivates (65), flavones and flavonols (62), organic acid and its derivatives (62), fatty acyls (47), phospholipids (33), and terpenoids (29) (Figure 2a). There were obvious similarities in replicates of each sample, and there were differences among these samples, as shown by the principal component analysis results according to intensity values for metabolites, which indicate that samples could be distinguished and were appropriate for further analysis (Figure 2b).

A total of 372 differentially accumulated metabolites (DAMs) were identified in all comparisons (Tables S2 and S3). In PCA persimmon varieties with fruit development, the number of up-regulated metabolites increased gradually, and the number of down-regulated metabolites decreased gradually. In PCNA persimmon varieties, the number of up-regulated metabolites increased first and decreased later, and the metabolites varied greatly with astringent persimmon, which may be related to the accumulation of flavor substances. The number of different accumulated metabolites in PCA and PCNA persimmon fruit was the highest from the young fruit stage (S1) to the fruit expansion stage (S2), which may be related to the rapid growth of the fruit. Compared with PCNA persimmon, the number of different accumulated metabolites of PCA persimmon increased from S1 to S4, and the number of up-regulated metabolites and down-regulated metabolites was highest in the mature stage (S4) and turning stage (S3), respectively.

### 2.3. KEGG Enrichment Analysis of DAMs and Galactose Metabolism Pathway Analysis

KEGG enrichment analysis was used to classify the functions of the differentially accumulated metabolites identified in PCNA and PCA persimmon developing fruit (Table S3). These 372 DAMs were significantly enriched in 95 metabolic pathways, including ten major KEGG pathways, namely, metabolic pathways (160 metabolites), biosynthesis of secondary metabolites (83), biosynthesis of amino acids (32), ABC transporters (29), purine metabolism (24), pyrimidine metabolism (22), galactose metabolism (21), flavonoid biosynthesis (19), carbon metabolism (17), and starch and sucrose metabolism (10).

**Figure 3:**
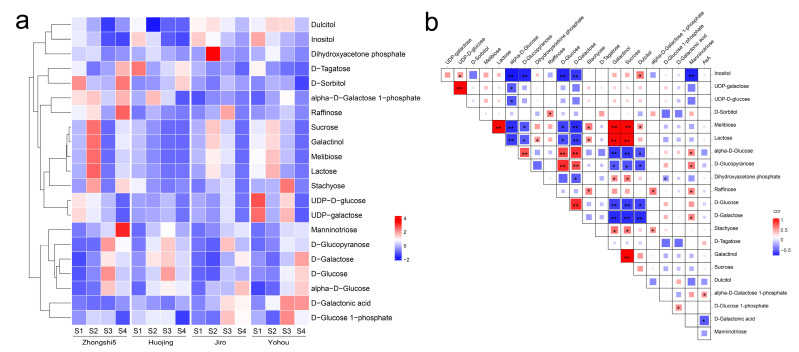
Overview of galactose metabolism pathways involving DAMs in four development stages of PCNA (‘Jiro’ and ‘Youhou’) and PCA (‘Zhongshi5’ and ‘Huojing’) fruit. (**a**) Heatmap of galactose metabolism pathways involving DAMs. (**b**) Pearson correlation of galactose metabolism pathways involving DAMs. * and ** show significant correlation in *p* < 0.05 and *p* < 0.01, respectively.

A total of 21 galactose metabolism-related DAMs were enriched, and DAMs associated with the myo-inositol pathway, the L-gulose pathway, and the D-galacturonate pathway were not enriched. The galactose metabolism pathway was significantly enriched in the comparison groups of Zhongshi5_S3 vs. Zhongshi5_S2, Zhongshi5_S3 vs. Jiro_S3, Zhongshi5_S3 vs. Yohou_S3, Zhongshi5_S4 vs. Zhongshi5_S3, Zhongshi5_S4 vs. Jiro_S4, Zhongshi5_S4 vs. Yohou_S4, Jiro_S3 vs. Jiro_S2, and Jiro_S4 vs. Jiro_S3. As shown in Figure 3a, the concentration of Lactose, D-Tagatose, and D-Sorbitol in PCA was higher than that of PCNA persimmon, while the concentration of Inositol, D-Glucose 1-photo, D-Galactose, and D-Galactonic acid was lower than that of PCNA persimmon. Figure 3b shows the findings of a Pearson correlation analysis performed on the content of DAMs related to the galactose metabolism of fruit in PCNA and PCA persimmon developing fruit. The AsA content was positively correlated with alpha-D-Galactose 1-phosphate content (*p* < 0.05) and was significantly negatively correlated with D-Galactonic acid content (*p* < 0.05). These results suggest that galactose metabolism is the major biosynthetic pathway of AsA in persimmon fruit. There were significant differences in AsA and galactose pathway-related DAM content in developing PCA and PCNA fruit.

### 2.4. Transcriptomic Analysis of PCA and PCNA Persimmon Developing Fruit

**Figure 4:**
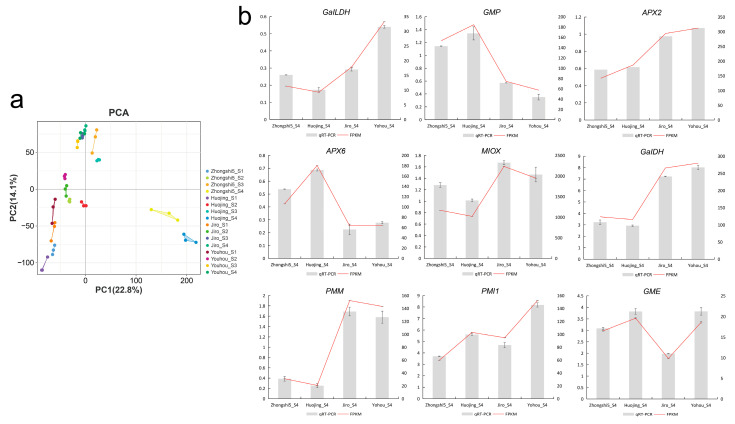
Overview of the RNA-seq data of persimmon developing fruit. (**a**) Principal component analysis. (**b**) The qRT-PCR relative gene expression and FPKM values of nine candidate genes in PCA (‘Zhongshi5’ and ‘Huojing’) and PCNA (‘Jiro’ and ‘Yohou’) cultivars at the mature stage (S4).

To identify the mRNA expression profiles in persimmon fruit, 48 RNA libraries from four developmental phases of four cultivars’ fruit were collected in triplicate. After removing adaptors and low-quality reads, the transcriptome yielded 321.44 GB of clean data. Each filtered sample consisted of 7.0 GB of high-quality data with an average Q30 base percentage of 92.42%. All clean reads were subsequently mapped onto the *D. kaki* genome; the total mapped ratio was 98.11% on average, and 4416 novel genes were identified in the transcriptome data. A principal component analysis based on the FPKM values was carried out. Our results show that the samples from the same stage of different persimmon varieties formed a single clade, and each sample was tightly grouped with its replicates, indicating that the samples could be clearly distinguished and were appropriate for further analysis (Figure 4a). Materials show apparent separation between PCA (‘Zhongshi5’ and ‘Huojing’) and PCNA (‘Jiro’ and ‘Yohou’) varieties fruit at four developing stages, indicating that significant differences were exhibited in different astringency persimmon fruit.

Differentially expressed genes (DEGs) in PCA and PCNA persimmon developing fruit were screened (Table S4). In PCA persimmon varieties, the number of DEGs increased with fruit development. The number of DEGs decreased gradually in PCNA persimmon fruit. The number of differentially expressed genes in PCA and PCNA fruit was higher in the turning stage (S3) and the mature stage (S4). Thus, S3 and S4 may be the critical stages of fruit flavor differentiation between PCA and PCNA persimmon fruit.

To confirm the accuracy of the RNA-seq data, nine AsA biosynthesis and recycling genes were verified by qRT-PCR. For these genes, the qRT-PCR relative gene expression matched the trends in the RNA-seq FPKM values, confirming the reliability of the RNA-seq data (Figure 4b).

### 2.5. Analysis of the AsA Biosynthesis and Recycling Pathway Genes

**Figure 5:**
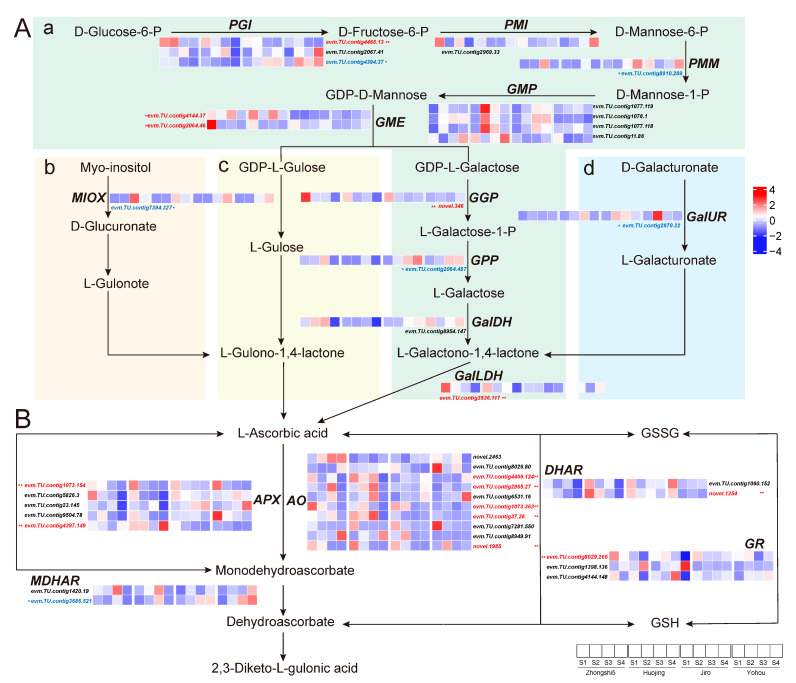
Expression patterns of 17 genes involved in (**A**) AsA biosynthesis and (**B**) AsA recycling. AsA biosynthesis pathways include (**a**) the L-galactose pathway, (**b**) the myo-inositol pathway, (**c**) the L-gulose pathway, and (**d**) the D-galacturonate pathway. The heatmap represents the normalized FPKM values by Z-score. * and ** show significant correlation in *p* < 0.05 and *p* < 0.01, respectively.

To further investigate the AsA content variation between PCA and PCNA persimmon fruit, expression patterns of AsA biosynthesis genes were analyzed in developing fruit (Figure 5A). A total of 17 AsA biosynthesis genes (FPKM ≥ 1 in at least one sample) were detected as expressed genes, including *PGI*, *PMI*, *PMM*, and *GMP* in the early L-galactose pathway, *GME*, *GGP*, *GPP*, *GalDH*, and *GalLDH* genes involved in the final five synthesis steps in the L-galactose pathway, *MIOX* in the MI pathway, and *GalUR* in the D-galacturonate pathway. In the L-galactose pathway, the expression of *PGI* (*evm.TU.contig4466.13*), *GME* (*evm.TU.contig4144.37* and *evm.TU.contig2064.46*), *GGP* (*novel.346*), and *GalLDH* (*evm.TU.contig3536.117*) genes shows a remarkably positive correlation with AsA content (*p* < 0.01). In contrast, *PGI* (*evm.TU.contig4394.37*), *PMM* (*evm.TU.contig8910.289*), and *GPP* (*evm.TU.contig2064.487*) exhibit a significantly negative correlation with AsA content (*p* < 0.05). In the D-galacturonate pathway and MI pathway, the correlation coefficients between the *GalUR* (*evm.TU.contig2970.22*), *MIOX* (*evm.TU.contig7394.327*) genes and AsA content were −0.304 and −0.295, respectively, and there was a significant negative correlation (*p* < 0.05).

Among these differentially expressed genes in the AsA biosynthesis pathway, the *GME* (*evm.TU.contig4144.37*) gene exhibits the highest correlation with ascorbic acid content (r = 690, **, *p* < 0.01). The expression of this *GME* gene was higher in PCA-type than in PCNA-type fruit in the young fruit stage, fruit expansion stage, and the turning stage, but not in the mature stage, which is consistent with the results that ascorbic acid content was significantly higher in PCA than in PCNA fruit in S1–S3. The results show that the *GME* (*evm.TU.contig4144.37*) gene may be the critical gene for ascorbic acid differences in PCA and PCNA persimmon fruit.

AsA recycling also played an important role in maintaining AsA contents. Thus, the expression levels of genes involved in this pathway were also detected, including *APX* (5 genes), *AO* (10), *MDHAR* (2), *DHAR* (2), and *GR* (3). The correlation coefficients between *AO* (*evm.TU.contig2965.27*, *evm.TU.contig1073.363*, *novel.1985*, *evm.TU.contig4466.124*, and *evm.TU.contig37.26*) gene expression and AsA content were 0.375, 0.481, 0.496, and 0.660, respectively, and show a strongly positive correlation (*p* < 0.01). The AsA content was significantly positively correlated with *APX* genes (*evm.TU.contig1073.154* and *evm.TU.contig4397.149*); their correlation coefficients were 0.405 and 0.357, respectively (*p* < 0.01). The expression of glutathione cycle (GSSG/GSH) genes *DHAR* (*novel.1254*) and *GR* (*evm.TU.contig8029.265*) also shows a significantly positive correlation (*p* < 0.01) with AsA content. In contrast, the expression levels of *MDHAR* show a negative correlation (*p* < 0.05) with AsA content in persimmon fruit. Overall, there was a strong positive correlation between AsA content and genes involved in AsA recycling, while only the *MDHAR* gene exhibits a negative correlation (Figure 5B).

### 2.6. Weighted Gene Co-Expression Network (WGCNA)

**Figure 6:**
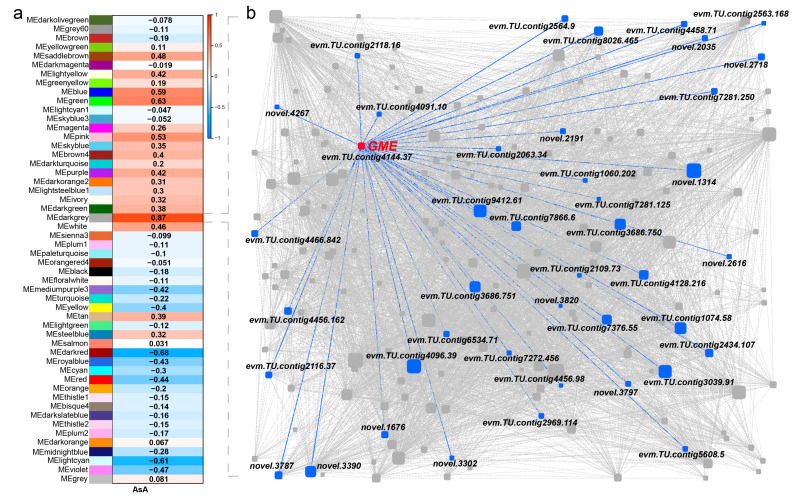
Weighted gene co-expression network analysis. (**a**) Gene modules were defined using WGCNA and their association with AsA content. The numbers in the heatmap show the correlation. (**b**) The linkages of AsA in the dark gray module. The circles were sized by gene connectivity.

To identify the gene regulatory networks associated with the AsA content of persimmon fruit, we analyzed the correlation relationships between gene expression and AsA content using WGCNA. WGCNA analysis was performed using 25,589 genes, and 51 merged co-expression gene modules were identified. Only the dark gray module shows a significant positive correlation with AsA content (r = 0.87, *p* = 8 × 10^−16^) (Figure 6a). Further analysis revealed that 198 genes and 266 TFs related to AsA content were identified in the module.

The highly connected genes of the darkgrey module were further investigated as potential key factors related to persimmon AsA content variation. Based on the degree of connectivity (kWithin value), 10 genes (top 10%) were defined as candidate hub genes. The gene co-expression network identified one orthologue of *GME* (*evm.TU.contig4144.37*) in the L-galactose pathway, showing high expression in fruit and a close correlation with the AsA content. Four hub genes were identified as potential regulators of AsA content, displaying connectivity with the *GME* gene, including the *DNA methylation gene* (*evm.TU.contig9412.61*), *methyltransferase gene* (*evm.TU.contig3686.751*), *F-box* (*evm.TU.contig7376.55*), and *Actin-Like Protein 1* (*ALP1*, *novel.3390*). The *GME* gene also shows high relevance with the *methyltransferase gene* (*evm.TU.contig3686.750*), *TLC* (*evm.TU.contig2109.73*), and *WD repeat* (*evm.TU.contig7281.250*). The results suggest that the *GME* gene plays a more important regulatory role in the L-galactose pathway, and its co-expressed genes, especially DNA methylation and the methyltransferase gene, might also be involved in regulating AsA synthesis (Figure 6b).

## 3. Discussion

Ascorbic acid is a crucial antioxidant that widely exists in plants [29], and the human body cannot synthesize AsA because of a lack of the L-guluronic acid-1,4-lactone oxidase enzyme [30]. Therefore, AsA produced by plants has many benefits for human health [31]. In addition, AsA also plays a vital role in plant resistance to abiotic stress, such as drought, salinity, and freezing [32,33]. Persimmon fruit is rich in bioactive compounds, of which the tannin and carotenoid variations are well-known [23,34], yet little is known about the ascorbic acid in persimmon fruit. This study chose four typical persimmon varieties to comprehensively analyze the reason for AsA variation among persimmon fruit. We investigated the AsA content and expression of genes related to AsA synthesis and recycling during fruit development. This investigation has excellent value in improving the quality and stress resistance of persimmon fruit, providing a basis for generating new varieties of persimmon with high AsA through molecular breeding techniques, thus increasing the commercial value and market competitiveness of persimmon fruit.

Ascorbic acid is one of the essential components of fruit nutritional quality [35]. Some horticultural plants contain high levels of AsA in fruit, for example, bananas (18.6 mg), blackberries (21.0 mg), lemons (74.3 mg), and oranges (83.2 mg) [36]. The results of this study show that ‘Zhongshi5’ (104.49 mg), ‘Huojing’ (48.69 mg), ‘Jiro’ (69.69 mg), and ‘Yohou’ (47.48 mg) persimmon fruit contained highly ascorbic acid content at maturity, which is consistent with Seung et al. (2000) [36]. The price of persimmon is more inexpensive than the other fruits; adults need 75–90 mg of vitamin C each day [37]. Thus, a persimmon a day is sufficient for human daily intake. In addition, the AsA content of ‘Huojing’ fruit is as high as 1211.7 mg/100 g at the first stage. The physiological fruit drop of persimmon mainly occurred at the early fruit development stage and the later stages of fruitlet growth [38,39], and the results of this study show that persimmon contained high AsA content in the young fruit stage. The utilization of ascorbic acid in the young fruit droppings will effectively improve the persimmon industry and production value, thus increasing the motivation for cultivating persimmon.

AsA accumulation is a complex and compound biological process in plants, containing AsA synthesis and AsA recycling [11]. The L-Gal pathway is the major biosynthetic pathway of AsA in higher plants [13,40], like cabbage [41], peach [42], and tomato [43]. The other alternative pathways, including the myo-inositol pathway, L-gulose pathway, and D-galacturonate pathway, also supported AsA biosynthesis in plants [11]. In addition to the above biosynthetic pathway, regeneration of AsA via the Foyer-Halliwell-Asada cycle is also a method for AsA production [44,45]. In this study, five L-Gal pathway genes and eight recycling pathway genes were found to be significantly correlated with ascorbic acid content in persimmon fruit (*p* < 0.01). It can be seen that the L-Gal biosynthesis pathway plays a significant role in AsA biosynthesis and that the recycling pathway also contributes to the variation in ascorbic acid content.

GDP-D-mannose 3,5-epimerase (*GME*) is considered to be one of the major enzymes of plant ascorbate accumulation [46,47]. The *GME* enzyme catalyzes the epimerization of GDP-D-mannose to produce GDP-L-galactose in the L-galactose pathway and GDP-L-gulose in an alternative L-gulose pathway [48,49]. The function of *GME* has been demonstrated in higher plants such as tomatoes (Solanum lycopersicum), and the ascorbate content decreased significantly in GME-silenced tomatoes [50]. *MsGME* from alfalfa (*Medicago sativa*) can effectively enhance acid, drought, and salt tolerance in *GME*-overexpression Arabidopsis by increasing ascorbate accumulation [47]. In this study, the results show that the expression of the *GME* gene (*evm.TU.contig4144.37*) was higher in PCA persimmon fruit than in PCNA fruit in the first three periods, while there was no difference in expression in the last period, which was consistent with the pattern that AsA content was significantly higher in PCA than in PCNA fruit from S1 to S3. The *GME* gene (*evm.TU.contig4144.37*) may be an essential gene for flavor differences in ascorbic acid in PCA and PCNA persimmon fruit.

## 4. Materials and Methods

### 4.1. Plant Materials

Fruit of PCNA type (varieties ‘Yohou’ and ‘Jiro’) and PCA type (varieties ‘Huojing’ and ‘Zhongshi5’) persimmon were harvested in Yuanyang County, Henan Province, China (34°55′18″–34°56′27″ N, 113°46′14″–113°47′35″ E). The cultivation and management of these 10-year-old cultivars were consistent, and these trees were placed with a row spacing of 3 × 4 m. The persimmon fruit was sampled at four typical developing stages, including the young fruit stage (S1, fruit size reached 40% of final size), fruit expansion stage (S2, fruit size reached 70% of final size), turning stage (S3, initial change in fruit color), and mature stage (S4, fruit color fully developed and astringency loss). The persimmon fruit of four varieties was randomly selected and collected from three clones; each replicate was composed of ten fruits. Fruit fresh in the equatorial plane was immediately frozen in liquid nitrogen, stored at −80 °C, and used for further RNA extraction and metabolite detection.

### 4.2. Extraction and Determination of AsA

Approximately 1 g of fruit was ground into powder, extracted with 5 mL of a 5% (*w*/*v*) trichloroacetic acid (TCA) solution with ultrasound for 20 min, and centrifuged at 8000 r/min for 20 min. The supernatant is the extract for AsA. After that, 200 µL of the extract for AsA was added to 1.80 mL of distilled water for dilution. Next, 1.0 mL of the dilution, 1.0 mL of 5% (*w*/*v*) TCA, and 1.0 mL of anhydrous ethanol were added and fully mixed. Then, 0.5 mL of 0.4% phosphoric acid-ethanol, 1.0 mL of 0.5% BP-ethanol, and 0.5 mL of 0.03% FeCl_3_-ethanol were added and mixed to a total volume of 5.0 mL. The mixture was heated at 30 °C for 60 min and vortexed every 5 min. The AsA content was calculated based on the absorbance of the L-ascorbic acid standard (SV8120, Beijing Solarbio Science and Technology, Beijing, China) at 534 nm, and three biological and technical replicates were conducted.

### 4.3. Transcriptome Data Analysis

Total RNA was extracted using the TRIzol Total RNA Isolation Kit (Sangon, Shanghai, China), and a library was established. The integrity of RNA was examined using the Bioanalyzer 2100. The sequencing of the NovaSeq platform (Illumina, San Diego, CA, USA) generated 150 bp paired-end readings. The filtered reads were mapped to the *D. kaki* reference genome [51] using HISAT2 software [52]. The StringTie software was used to predict new transcripts [53]. The FeatureCounts software [54] calculated the number of reads mapped to each gene. The gene FPKM value was calculated based on the length of the gene and the count of reads mapped to it. The online software Hiplot (https://hiplot.cn/basic/heatmap) [55] was used to prepare heatmaps, accessed on 20 June 2023. Differentially expressed genes (DEGs) were screened according to the |log2-fold change| and the padj value, with thresholds of |log2-fold change| ≥ 1.0, and padj ≤ 0.05. The weighted gene co-expression network analysis was prepared using all expressed genes and analyzed using the R package WGCNA [56]. The co-expression networks were visualized using online OmicShare tools (www.omicshare.com/tools), accessed on 6 December 2022. Transcriptome analysis includes a re-analysis of ‘Jiro’ and ‘Huojing’ transcriptomic data (Bioproject ID PRJNA910302), along with new ‘Yohou’ and ‘Zhongshi5’ transcriptomic data (PRJNA989105).

### 4.4. Metabolome Data Analysis

Metabolite detection was conducted following the methods of Wang et al. [19] and Meng et al. [57]. Persimmon fruit was freeze-dried, weighted (100 mg), and extracted using 1.0 mL of 70% aqueous methanol. Metabolite detection was performed using the ExionLC™ AD system (SCIEX, Framingham, MA, USA) equipped with the QTRAP^®^6500+ mass spectrometer (SCIEX, USA). The injection volume for persimmon fruit extraction was 1.5 μL. Chromatographic separation was conducted using the Xselect HSS T3 column (2.1 × 150 mm, 2.5 μm, Waters, Milford, MA, USA) at a flow rate of 0.4 mL/min with the following mobile phases: 0.1% formic acid in water (solvent A) and 0.1% formic acid-acetonitrile (solvent B). The gradient program of solvent A/solvent B was set as follows: 0–2 min, 2% B; 2–15 min, 2% to 100% B; 15–17 min, 100% B; 17–17.1 min, 100% to 2% B; 17.1~20 min, 2% B.

The qualitative analysis of metabolites was carried out using Novogene’s in-house database based on secondary spectral information. The quantification of metabolites was conducted using multiple reaction monitoring (MRM) conditions. The KEGG database (Kyoto Encyclopedia of Genes and Genomes) was used to annotate metabolites [58]. Differentially accumulated metabolites (DAMs) were screened based on the VIP values, fold change (FC), and *p*-value, with thresholds of VIP ≥ 1.0, FC ≥ 1.5, or FC ≤ 0.667, and *p*-value ≤ 0.05.

### 4.5. Gene Expression Analysis by qRT-PCR

Nine AsA biosynthesis and recycling genes were randomly selected for qRT-PCR analysis. The primers for these genes are shown in Table S5. The qRT-PCR reaction program was conducted using the following conditions: 95 °C for 30 s, 40 cycles at 95 °C for 5 s, and followed by 55 °C for 25 s. The mean gene expression value was calculated according to the value of three biological replicates. The relative gene expression was calculated using the 2^−∆∆Ct^ method. The *GAPDH* gene [59] was used as the reference gene to normalize the targeted gene in persimmon.

## 5. Conclusions

In conclusion, the comprehensive metabolomic and transcriptomic analyses of typical PCNA varieties (‘Jiro’ and ‘Yohou’) and PCA varieties (‘Zhongshi5’ and ‘Huojing’) persimmon fruit were conducted. Persimmon fruit is rich in ascorbic acid, and fruit of the same astringency type persimmon exhibited a similar AsA accumulation pattern. AsA content was significantly higher in PCA persimmon fruit than PCNA fruit at the first three stages. The content of galactose pathway-related metabolites was significantly different in developing PCA and PCNA fruit. Combined gene expression and WGCNA analyses show that the expression of the *GME* (*evm.TU.contig4144.37*) gene was higher in PCA-type in S1–S3 and exhibited the highest correlation with AsA content. Four hub genes, including the *DNA methylation gene*, *methyltransferase gene*, *F-box*, and *Actin-like Protein*, were identified as potential regulators of the *GME* gene. These results will provide basic information on how persimmon astringency types affect AsA accumulation and useful information for further investigation on AsA content variation in persimmon fruit.

## Data Availability

The transcriptome sequencing raw data were deposited in the National Center for Biotechnology Information Sequence Read Archive (NCBI SRA) under the Bioproject ID PRJNA989105.

## Supplementary Materials

The following supporting information can be downloaded at: https://www.mdpi.com/article/10.3390/ijms242015362/s1.

## References

[1] Li M., Ma F., Zhang M., Pu F. (2008). Distribution and metabolism of ascorbic acid in apple fruits (*Malus domestica* Borkh cv. Gala). Plant Sci..

[2] Smirnoff N. (2018). Ascorbic acid metabolism and functions: A comparison of plants and mammals. Free Radic. Biol. Med..

[3] Shenoy N., Creagan E., Witzig T., Levine M. (2018). Ascorbic Acid in Cancer Treatment: Let the Phoenix Fly. Cancer Cell.

[4] Santos K., Bragança V., Pacheco L., Ota S., Aguiar C., Borges R. (2021). Essential features for antioxidant capacity of ascorbic acid (vitamin C). J. Mol. Model..

[5] Gallie D.R. (2013). L-Ascorbic Acid: A Multifunctional Molecule Supporting Plant Growth and Development. Scientifica.

[6] Pastori G., Kiddle G., Antoniw J., Bernard S., Veljovic-Jovanovic S., Verrier P., Noctor G., Foyer C. (2003). Leaf Vitamin C Contents Modulate Plant Defense Transcripts and Regulate Genes That Control Development through Hormone Signaling. Plant Cell.

[7] Maruta T., Ichikawa Y., Mieda T., Takeda T., Tamoi M., Yabuta Y., Ishikawa T., Shigeoka S. (2010). The Contribution of Arabidopsis Homologs of l-Gulono-1,4-lactone Oxidase to the Biosynthesis of Ascorbic Acid. Biosci. Biotechnol. Biochem..

[8] Visser D., Jansen N., Ijland M., de Koning T., van Hasselt P. (2011). Intracranial bleeding due to vitamin K deficiency: Advantages of using a pediatric intensive care registry. Intens. Care Med..

[9] Chaturvedi S., Khan S., Bhunia R., Kaur K., Tiwari S. (2022). Metabolic engineering in food crops to enhance ascorbic acid production: Crop biofortification perspectives for human health. Physiol. Mol. Biol. Pla..

[10] Davey M., Montagu M., Inzé D., Sanmartin M., Kanellis A., Smirnoff N., Benzie I., Strain J., Favell D., Fletcher J. (2000). Plant L-ascorbic acid: Chemistry, function, metabolism, bioavailability and effects of processing. J. Sci. Food Agric..

[11] Li H., Huang W., Wang G., Wang W., Cui X., Zhuang J. (2017). Transcriptomic analysis of the biosynthesis, recycling, and distribution of ascorbic acid during leaf development in tea plant (*Camellia sinensis* (L.) O. Kuntze). Sci. Rep..

[12] Mellidou I., Koukounaras A., Chatzopoulou F., Kostas S., Kanellis A.K. (2017). Plant Vitamin C: One Single Molecule with a Plethora of Roles. Fruit and Vegetable Phytochemicals.

[13] Wheeler G., Jones M., Smirnoff N. (1998). The biosynthetic pathway of vitamin C in higher plants. Nature.

[14] Ishikawa T., Dowdle J., Smirnoff N. (2006). Progress in manipulating ascorbic acid biosynthesis and accumulation in plants. Physiol. Plant..

[15] Wang Z., Xiao Y., Chen W., Tang K., Zhang L. (2010). Increased Vitamin C Content Accompanied by an Enhanced Recycling Pathway Confers Oxidative Stress Tolerance in Arabidopsis. J. Integr. Plant Biol..

[16] Agius F., González-Lamothe R., Caballero J.L., Muñoz-Blanco J., Botella M.A., Valpuesta V. (2003). Engineering increased vitamin C levels in plants by overexpression of a D-galacturonic acid reductase. Nat. Biotechnol..

[17] Jimenez A., Creissen G., Kular B., Firmin J., Robinson S., Verhoeyen M., Mullineaux P. (2002). Changes in oxidative processes and components of the antioxidant system during tomato fruit ripening. Planta.

[18] Pateraki I., Sanmartin M., Kalamaki M., Gerasopoulos D., Kanellis A. (2004). Molecular characterization and expression studies during melon fruit development and ripening of l-galactono-1,4-lactone dehydrogenase. J. Exp. Bot..

[19] Wang Y., Suo Y., Han W., Li H., Wang Z., Diao S., Sun P., Fu J. (2023). Comparative transcriptomic and metabolomic analyses reveal differences in flavonoid biosynthesis between PCNA and PCA persimmon fruit. Front. Plant Sci..

[20] Saleem M., Ejaz S., Anjum M., Nawaz A., Naz S., Hussain S., Ali S., Canan İ. (2020). Postharvest application of gum arabic edible coating delays ripening and maintains quality of persimmon fruits during storage. J. Food Process Preserv..

[21] Suo Y., Sun P., Cheng H., Han W., Diao S., Li H., Mai Y., Zhao X., Li F., Fu J. (2020). A high-quality chromosomal genome assembly of Diospyros oleifera Cheng. GigaScience.

[22] Han W., Wang Y., Li H., Diao S., Suo Y., Li T., Sun P., Li F., Fu J. (2023). Transcriptome and Metabolome Reveal Distinct Sugar Accumulation Pattern between PCNA and PCA Mature Persimmon Fruit. Int. J. Mol. Sci..

[23] Akagi T., Ikegami A., Suzuki Y., Yoshida J., Yamada M., Sato A., Yonemori K. (2009). Expression balances of structural genes in shikimate and flavonoid biosynthesis cause a difference in proanthocyanidin accumulation in persimmon (*Diospyros kaki* Thunb.) fruit. Planta.

[24] Yang X., Kui L., Tang M., Li D., Wei K., Chen W., Miao J., Dong Y. (2020). High-Throughput Transcriptome Profiling in Drug and Biomarker Discovery. Front. Genet..

[25] Fiehn O. (2002). Metabolomics—The link between genotypes and phenotypes. Funct. Genom..

[26] Yang C., Fang X., Wu X., Mao Y., Wang L., Chen X. (2012). Transcriptional Regulation of Plant Secondary Metabolism. F. J. Integr. Plant Biol..

[27] Han M., Cui R., Wang D., Huang H., Rui C., Malik W.A., Wang J., Zhang H., Xu N., Liu X. (2023). Combined transcriptomic and metabolomic analyses elucidate key salt-responsive biomarkers to regulate salt tolerance in cotton. BMC Plant Biol..

[28] Liu Y., Zhang W., Elango D., Liu H., Jin D., Wang X., Wu Y. (2023). Metabolome and Transcriptome Analysis Reveals Molecular Mechanisms of Watermelon under Salt Stress. Environ. Exp. Bot..

[29] Smirnoff N., Wheeler G. (2000). Ascorbic Acid in Plants: Biosynthesis and Function. Crit. Rev. Biochem. Mol..

[30] Dun W., Wei X., Wang L., Liu J., Zhao J., Sun P., Fang C., Xie X. (2023). Over-expression of *FaGalLDH* Increases Ascorbic Acid Concentrations and Enhances Salt Stress Tolerance in Arabidopsis thaliana. J. Plant Biol..

[31] Di Matteo A., Sacco A., Anacleria M., Pezzotti M., Delledonne M., Ferrarini A., Frusciante L., Barone A. (2010). The ascorbic acid content of tomato fruits is associated with the expression of genes involved in pectin degradation. BMC Plant Biol..

[32] Xu Y., Huang B., Hossain M., Munné-Bosch S., Burritt D., Diaz-Vivancos P., Fujita M., Lorence A. (2017). Exogenous Ascorbic Acid Mediated Abiotic Stress Tolerance in Plants. Ascorbic Acid in Plant Growth, Development and Stress Tolerance.

[33] Sah S.K., Reddy K.R., Li J. (2016). Abscisic Acid and Abiotic Stress Tolerance in Crop Plants. Front. Plant Sci..

[34] Qi Y., Liu X., Zhang Q., Wu H., Yan D., Liu Y., Zhu X., Ren X., Yang Y. (2019). Carotenoid accumulation and gene expression in fruit skins of three differently colored persimmon cultivars during fruit growth and ripening. Sci. Hortic..

[35] Vincente A.R., Manganaris G.A., Ortiz C.M., Sozzi G.O., Crisosto C.H., Florkowski W., Shewfelt R., Brueckner B., Prussia S. (2014). Chapter 5-Nutritional Quality of Fruits and Vegetables. Postharvest Handling.

[36] Lee S.K., Kader A.A. (2000). Preharvest and postharvest factors influencing vitamin C content of horticultural crops. Postharvest Biol. Tec..

[37] Harrison F.E. (2012). A critical review of vitamin C for the prevention of age-related cognitive decline and Alzheimer’s disease. J. Alzheimer’s Dis.

[38] George A., Mowat A., Collins R., Morley-Bunker M. (1997). The pattern and control of reproductive development in non-astringent persimmon (*Diospyros kaki* L.): A review. Sci. Hortic..

[39] Shaya F., David I., Yitzhak Y., Izhaki A. (2019). Hormonal interactions during early physiological partenocarpic fruitlet abscission in persimmon (*Diospyros Kaki* Thunb.) ‘Triumph’ and ‘Shinshu’ cultivars. Sci. Hortic..

[40] Linster C., Clarke S. (2008). L-Ascorbate biosynthesis in higher plants: The role of *VTC2*. Trends Plant Sci..

[41] Ren J., Chen Z., Duan W., Song X., Liu T., Wang J., Hou X., Li Y. (2013). Comparison of ascorbic acid biosynthesis in different tissues of three non-heading Chinese cabbage cultivars. Plant Physiol. Biochem..

[42] Imai T., Ban Y., Terakami S., Yamamoto T., Moriguchi T. (2009). L-Ascorbate biosynthesis in peach: Cloning of six l-galactose pathway-related genes and their expression during peach fruit development. Physiol. Plant..

[43] Li X., Ye J., Munir S., Yang T., Chen W., Liu G., Zheng W., Zhang Y. (2019). Biosynthetic Gene Pyramiding Leads to Ascorbate Accumulation with Enhanced Oxidative Stress Tolerance in Tomato. Int. J. Mol. Sci..

[44] Kunert K.J., Foyer C.H., Mittler R.O.N., Breusegem F.V. (2023). Chapter Three-The ascorbate/glutathione cycle. Advances in Botanical Research.

[45] Blauer J., Kumar G., Knowles L., Dhingra A., Knowles N. (2013). Changes in ascorbate and associated gene expression during development and storage of potato tubers (*Solanum tuberosum* L.). Postharvest Biol. Tec..

[46] Mounet-Gilbert L., Dumont M., Ferrand C., Bournonville C., Monier A., Jorly J., Lemaire-Chamley M., Mori K., Atienza I., Hernould M. (2016). Two tomato GDP-D-mannose epimerase isoforms involved in ascorbate biosynthesis play specific roles in cell wall biosynthesis and development. J. Exp. Bot..

[47] Ma L., Wang Y., Liu W., Liu Z. (2014). Overexpression of an alfalfa GDP-mannose 3, 5-epimerase gene enhances acid, drought and salt tolerance in transgenic Arabidopsis by increasing ascorbate accumulation. Biotechnol. Lett..

[48] Major L., Wolucka B., Naismith J. (2005). Structure and Function of GDP-Mannose-3′,5′-Epimerase:  An Enzyme which Performs Three Chemical Reactions at the Same Active Site. J. Am. Chem. Soc..

[49] Beerens K., Gevaert O., Desmet T. (2022). GDP-Mannose 3,5-Epimerase: A View on Structure, Mechanism, and Industrial Potential. Front. Mol. Biosci..

[50] Gilbert L., Alhagdow M., Nunes-Nesi A., Quemener B., Guillon F., Bouchet B., Faurobert M., Gouble B., Page D., Garcia V. (2009). GDP-d-mannose 3,5-epimerase (GME) plays a key role at the intersection of ascorbate and non-cellulosic cell-wall biosynthesis in tomato. Plant J..

[51] Li H., Sun P., Wang Y., Zhang Z., Yang J., Suo Y., Han W., Diao S., Li F., Fu J. (2023). Allele-aware chromosome-level genome assembly of the autohexaploid *Diospyros kaki* Thunb. Sci. Data.

[52] Zhang Y., Park C., Bennett C., Thornton M., Kim D. (2021). Rapid and accurate alignment of nucleotide conversion sequencing reads with HISAT-3N. Genome Res..

[53] Shumate A., Wong B., Pertea G., Pertea M. (2022). Improved transcriptome assembly using a hybrid of long and short reads with StringTie. PLOS Comput. Bio..

[54] Liao Y., Smyth G.K., Shi W. (2014). FeatureCounts: An efficient general purpose program for assigning sequence reads to genomic features. Bioinformatics.

[55] Li J., Miao B., Wang S., Dong W., Xu H., Si C., Wang W., Duan S., Lou J., Bao Z. (2022). Hiplot: A comprehensive and easy-to-use web service for boosting publication-ready biomedical data visualization. Brief. Bioinform..

[56] Langfelder P., Horvath S. (2008). WGCNA: An R package for weighted correlation network analysis. BMC Bioinform..

[57] Meng Y., Du Q., Du H., Wang Q., Wang L., Du L., Liu P. (2023). Analysis of chemotypes and their markers in leaves of core collections of Eucommia ulmoides using metabolomics. Front. Plant Sci..

[58] Kanehisa M., Goto S., Kawashima S., Okuno Y., Hattori M. (2004). The KEGG resource for deciphering the genome. Nucleic Acids Res..

[59] Du G., Wang L., Li H., Sun P., Fu J., Suo Y., Han W., Diao S., Mai Y., Li F. (2019). Selection and validation of reference genes for quantitative gene expression analyses in persimmon (*Diospyros kaki* thunb.) using real-time quantitative PCR. Biol. Fu..

